# Devolving countdown to countries: using global resources to support regional and national action

**DOI:** 10.1186/s12889-016-3400-7

**Published:** 2016-09-12

**Authors:** Zulfiqar A. Bhutta, Mickey Chopra

**Affiliations:** 1 Centre for Global Child Health, The Hospital for Sick Children, 686 Bay Street, Toronto, M5G 0A4 ON Canada; 2 Center of Excellence in Women and Child Health, the Aga Khan University, Karachi, Pakistan; 3 Dalla Lana School of Public Health, University of Toronto, Toronto, Canada; 4 The World Bank, Washington DC, 20433 USA

## Background

As the world embarks on the quest to achieve the sustainable development goals (SDGs), building on the momentum and lessons of the millennium development goals (MDGs), several aspects are clear. The SDGs are deliberately visionary and all-encompassing and in relation to health and nutrition, and include most of the social determinants of health. The health goal is also a much broader goal than the focus on maternal and child health and infectious diseases that was found in the MDGs. Notwithstanding the above, three key aspects of the health goal (SDG 3) related to maternal and child health stand out.

Firstly, achieving further gains in maternal and child health and survival cannot depend on the momentum of the past decade and will need concerted action and a focus on the bottlenecks and disparities highlighted previously [1]. Secondly, the renewed global strategy for every woman every child, The Global Strategy for Women’s, Children’s and Adolescents’ Health (Global Strategy), now includes several aspects of the continuum of care for women and children that were hitherto ignored. These include aspects of adolescent health, preconception care as well as child development outcomes. Lastly the focus on social determinants of health in the SDGs opens up huge opportunities for investments, multi-sectoral action and accountability.

The transition from the successful Countdown to 2015 (Countdown 2015) activities to Countdown 2030 (Countdown 2030) has taken a fair amount of consultation and thinking among the consortium partners, especially the academic groups, potential funders, UN and bilateral agencies. Although several accountability processes have been highlighted for the Global Strategy [2] with an independent accountability panel, the role of a continued Countdown like process received much support. However, it was also felt that a renewed Countdown 2030 would need to build on the successes of the past initiative and focus on bridging the gaps around areas that may have been less than optimal. In particular, much support was expressed for focusing on monitoring and evaluation of country level progress that went beyond the overall summative evaluations and country summaries that were a highlight of previous Countdown Reports [3–5].

During the tenure of Countdown 2015, various stakeholders felt a strong need to pivot the global level monitoring, analytical and accountability work to more actively support activities at country level in selected countries. The initial purpose of this work was to identify those that made important progress in reproductive, maternal, newborn and child health (RMNCH), and understand how such progress was achieved. A parallel objective was to build national capacity in the four areas of Countdown 2015 work: coverage monitoring, equity, financing, and health systems and policy, as well as in modeling of lives saved (using the Lives Saved Tool) with key interventions. More recently, Countdown 2015 country case-studies started to also focus on some countries where progress was slower than desired, in order to understand existing barriers and propose solutions. In particular there is much interest at policy and programmatic level in understanding inequities in coverage at sub-national level and determinants thereof, features that have been hallmarks of several recent country case studies [6–8]. Many of the studies highlighted in this special issue represent multi-stakeholder collaborations between academia, civic society organisations and UN agencies; they represent strong partnerships with the lead taken by a local organisation, frequently an academic body.

These case studies have been accompanied by considerable efforts in capacity enhancement and, in some instances, development of strong partnerships with the potential of continuing beyond the life span of the case studies themselves. Countdown 2015 undertook several training workshops in enhancing capacities in coverage estimation, assessment of inequities as well as measurements of health systems and policies. As Countdown 2030 develops there is much greater emphasis on devolution of work and capacities to countries with a hub and spoke model of regional hubs and centres with the capacity and potential of providing technical support for national level monitoring and evaluation. In the first instance regional hubs will be established to cover South-central Asia, Africa and Latin America. The regional hubs will be based in academic institutions in each region with a network of collaborating bodies reflecting the partner organisations of Countdown 2030. The aims of these hubs would be to coordinate a series of country and regional evaluations along the lines of Countdown 2015 but with enhanced focus on understanding regional and national time trends and disparities in intervention coverage, health policies, systems and services; and health financing. These hubs will support countries, technical institutions, civil society organisations, and partnerships in countries to carry out analyses, and will help conduct and publish cross-country analyses and comparisons. They will build national capacity in the four areas of the Countdown 2030 work: coverage monitoring, equity, financing, and health systems and policy, as well as in modeling of lives saved. The focus on regional, national and sub-national analyses will also provide important inputs to policies and initiatives that are at the core of the strategies to achieve SDG 3 such as universal health care and initiatives to achieve the goals of the decade of nutrition [9].

Other major initiatives to achieve change at country level include targeted multi-stakeholder efforts supported by the global Partnership for Maternal, Newborn and Child Health as well as the Global Financing Facility for RMNCH supported by the World Bank [10]. Countdown 2030 could provide important inputs to these initiatives as they begin to impact country level action and scale up.

Any measure of a sustainable future depends upon the welfare of women, adolescents, children and newborns. The chances of achieving the SDGs will depend upon accelerating and spreading the welfare gains made by many across the world. Accurately measuring and monitoring such gains especially for the most vulnerable and using this to hold duty bearers accountable are essential actions. The new Countdown 2030 has the potential to make an important contribution to such actions and must do so in the places where there are still preventable deaths and morbidity.
